# Supplementary material for: Functional specialization of the human posterior parietal cortex in visually and proprioceptively driven reaching corrections
Source: Commun Biol. 2025 Nov 24;8:1658. doi: 10.1038/s42003-025-09040-5 (PMC12644796; doi:10.1038/s42003-025-09040-5)
Supplement: Supplementary file 1 — Supplementary Information [file 42003_2025_9040_MOESM1_ESM.pdf]

Functional Specialization of the Human Posterior Parietal Cortex in Visually and Proprioceptively Driven Reaching Corrections

Brandolani Riccardo[1,2], Galletti Claudio[1], Di Gloria Davide[3], Fattori Patrizia[1,4], Breveglieri Rossella[1,4]

[1] Department of Biomedical and Neuromotor Sciences, University of Bologna, Piazza di Porta San Donato, 2, 40126 Bologna (Italy)

[2] University of Camerino, Center for Neuroscience, 62032 Camerino (Italy)

[3] STAM s.r.l., via Lorenzo Pareto 8AR, 16129, Genova (Italy)

[4] Interdepartmental center for industrial research-Aerospace, University of Bologna, Via Baldassarre Carnaccini 12, 47121 Forlì FC, Italy

*Corresponding author

Prof. Rossella Breveglieri

Phone: 00390512091890

Fax: 00390512091737

Email: rossella.breveglieri@unibo.it

Keywords: reaching, posterior parietal cortex, motor control, reaching corrections.

Supplementary methods

Procedures to estimate the resting motor threshold (rMT)

Motor evoked potentials (MEPs) induced by stimulation of the left primary motor cortex were recorded from the right first dorsal interosseous (FDI) muscle by means of a 2-channel DuoMAG MEP amplifier. Electromyography (EMG) signals were FIR-filtered and digitized at a sampling rate of 5 kHz. Pairs of disposable pre-gelled Ag–AgCl surface electrodes were placed in a belly tendon montage with a ground electrode on the midpoint of the palmar surface of the wrist. The optimal scalp position for inducing MEPs from the right FDI was first localized, and the rMT was determined from that position. The rMT was defined as the minimal intensity of stimulator output that produced MEPs with an amplitude of at least 50μV in the FDI with a probability of 50% [1].

Electromyographic (EMG) recordings

During each stimulation session, EMG was used to monitor muscle activity of the extensor carpi radialis (ECR), and of the flexor carpi radialis (FCR) muscles (Fig. 5B). Surface electromyograms were recorded by means of a Digitimer D440-4 system (Digitimer, Welwyn Garden City, UK), amplified to 1000x, with a sample rate of 1 kHz recorded using a Micro1401 data acquisition interface controlled by Signal software (version 7; Cambridge Electronic Design Ltd, Cambridge, UK), sorted on a computer for offline analysis.

Analysis of the EMG responses

EMG signals were recorded using the Signal software (see above) and exported into Matlab. EMG data were first rectified after removing the DC component. Since TMS was applied during movement execution at 50, 150, and 250 ms after movement onset, TMS artifacts appeared in the EMG recordings. To remove these artifacts, the affected portions of the EMG trace were deleted (approximately 10ms of trace), and the missing data were filled using the Matlab function *fillmissing*. To analyze the responses, we smoothed the EMG traces using a sliding window of 100 ms with a sample rate of 1 ms. Since we aimed to study how different types of trial affected the EMG recordings in the first portion of the movement, where an online correction may occur, we analyzed each EMG trace from 50 to 350 ms from the movement onset to include the entire stimulation epoch and well below the lowest average movement time across conditions. These 300ms traces provided valuable insights into how the different conditions influenced the EMG data. Specifically, we divided the 300ms traces into two epochs: early (first 150 ms) and late (last 150 ms). The magnitude of transient

EMG responses during these epochs was measured using an approximation of the EMG integral (cumulative sum: Matlab function *cumsum*)[2–4]. This method enabled us to compare the magnitude of EMG activity across all conditions for each muscle during the two epochs. Statistical comparisons were performed with four (one for each muscle in each experiment) 3-way repeated measures analysis of variance (ANOVA) on the EMG activity with TMS (3 levels, SHAM, V1/V2, hV6A for Experiment 1 and SHAM, IPS, hPEc for Experiment 2), Condition (4 levels, Unperturbed near, Unperturbed far, Visual perturbation, Proprioceptive perturbation) and Epoch (2 levels, Early, Late) as within-participants factors. This analysis on EMG activity was performed twice: during movement execution and prior to movement onset. Whenever sphericity was violated (Mauchly test, $p < 0.05$), we used the lower-bound correction.

Analysis of reaction times and movement times

Reaching onset and offset were identified as the moments when the velocity of the kinematic markers exceeded or dropped below 30 mm/s, respectively [5,6]. Reaction time was defined as the time interval between the go signal and the reaching onset. Movement time was obtained by subtracting the reaching onset from the respective reaching offset. Statistical comparisons were performed using 2-way repeated measures ANOVA on reaction times and movement times with TMS (3 levels, SHAM, V1/V2, hV6A for Experiment 1 and SHAM, IPS, hPEc for Experiment 2) and Condition (4 levels, Unperturbed far, Unperturbed near, Visual perturbation, Proprioceptive perturbation) as within-participants factors.

Analysis of reaching accuracy

Reaching accuracy was determined for each participant and stimulation condition by computing the Euclidean distance between the mean endpoint (the landing point of the index finger on the touch screen) and the corresponding target position [7]. The average x and y coordinates of the endpoints were calculated after converting the measurements from pixels to millimeters. Statistical comparisons were performed using 2-way repeated measures ANOVA on reaching accuracy with TMS (3 levels, SHAM, V1/V2, hV6A for Experiment 1 and SHAM, IPS, hPEc for Experiment 2) and Condition (4 levels, Unperturbed far, Unperturbed near, Visual perturbation, Proprioceptive perturbation) as within-participants factors.

Supplementary results

EMG activity, movement times, and reaction times validate our task design.

EMG signals were recorded as a sanity check to show the stronger force applied by the participants during perturbations triggered by the application of an external force. As expected, the EMG activity of ECR was significantly modulated by the interaction between condition and epoch in both Experiments 1 and 2 (all $F > 8.16$, all partial $\eta^2 > 0.4$, all $p < 0.01$; Fig. S1A-B left). Specifically, earlier in the movement, EMG activity was significantly higher in the proprioceptive perturbation condition compared to the others (both experiments, all $p < 0.0003$). Also during perturbations triggered by the shift of visual target a slightly stronger force was applied relatively to the other conditions, but later on and not consistently between experiments: in the late epoch EMG activity in the visual perturbation condition was higher than in all other conditions in Experiment 1 (all $p < 0.0008$) and all the other conditions but the proprioceptive perturbation one in Experiment 2 (all $p < 0.02$). The main effect of epoch was significant in both experiments (all $F > 5.25$, all partial $\eta^2 > 0.3$, all $p < 0.04$), with the early epoch having lower EMG activity than the late one (all $p < 0.04$), and the main effect of condition only in the second experiment ($F = 5.14$, partial $\eta^2 = 0.3$, $p = 0.04$), with the proprioceptive perturbation condition having higher EMG activity than all the others (all $p < 0.008$). All other effects were not significant (all $F < 4.2$, all partial $\eta^2 < 0.26$, all $p > 0.06$).

The trend described for ECR was also observed for FCR, with a significant interaction between condition and epoch (all $F > 8.35$, all partial $\eta^2 > 0.41$, all $p < 0.0002$; Fig. S1A-B right). During the early epoch, EMG activity in the proprioceptive perturbation condition was significantly higher than in the other conditions (all $p < 0.0001$). Additionally, there were also significant main effects of epoch in both experiments (all $F > 8.98$, all partial $\eta^2 > 0.43$, all $p < 0.01$), with the early epoch having lower EMG activity than the late one (all $p < 0.04$), and a main effect of condition only in the second experiment ($F = 13.2$, partial $\eta^2 = 0.52$, $p = 0.003$), with the proprioceptive perturbation condition having higher EMG activity than all the others (all $p < 0.0002$). All other effects, including those by TMS, were not significant (all $F < 3.1$, all partial $\eta^2 < 0.18$, all $p > 0.06$).

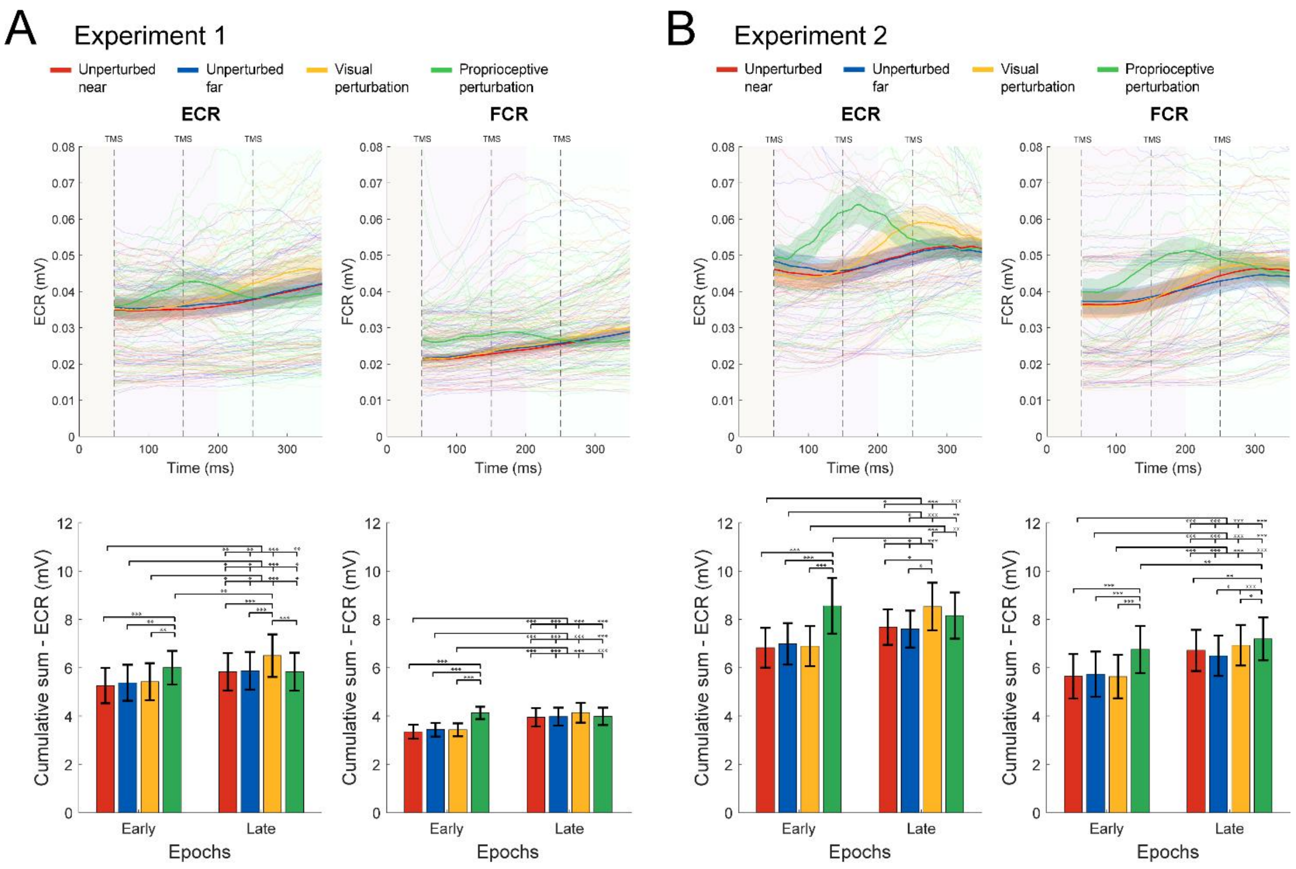

Figure S1. EMG activities of ECR and FCR muscles during movement execution: (A) Results from Experiment 1 (B) Results from Experiment 2. Top: Vertical lines represent the time occurrence of TMS pulses. Different colors represent different task conditions (red: unperturbed near, blue: unperturbed far, yellow: visual perturbation, green: proprioceptive perturbation) shaded areas represent ± one Standard Error of the Mean (SEM). The shaded rectangles represent early (light magenta) and late (light green) epochs. Bottom: cumulative sum of the EMG activities in different epochs and conditions. Error bars represent ± one SEM. Asterisks mark a statistically significant difference (* = $p < 0.05$, ** = $p < 0.01$, *** = $p < 0.001$). As no TMS effects were significant, we have collapsed the data from the different stimulation conditions.

Analysis of EMG activity before movement onset (Fig. S2) revealed no significant effects in either experiment (all $F < 2$, all partial $\eta^2 < 0.14$, all $p > 0.08$).

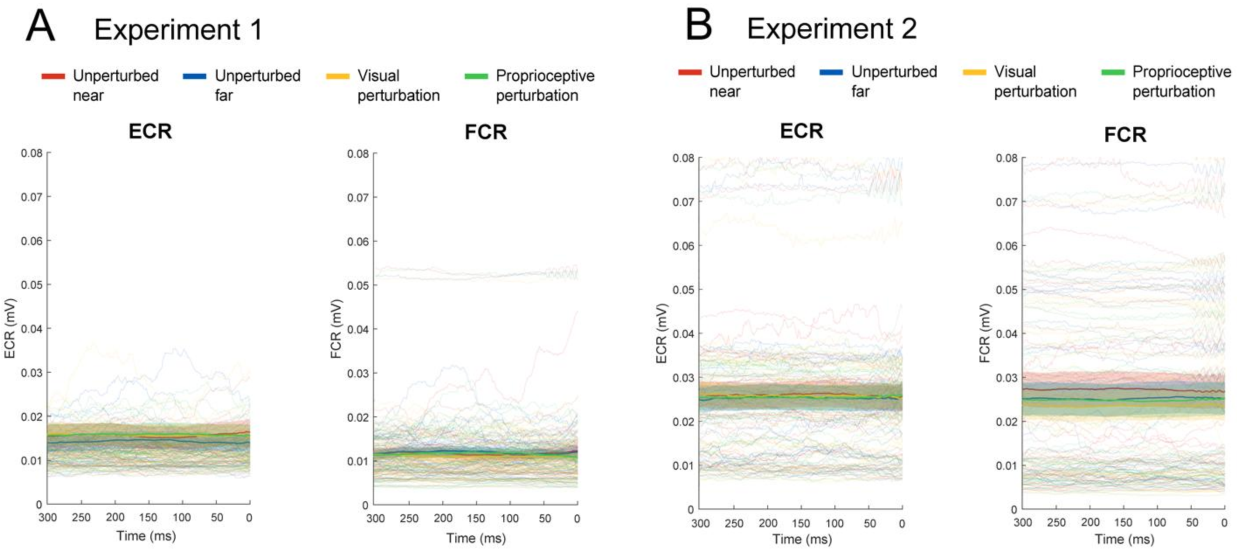

Figure S2. EMG activities of ECR and FCR muscles before movement onset: (A) Results from Experiment 1 (B) Results from Experiment 2. Conventions as in Figure S1.

Movement times were influenced by the condition in both experiments (all $F > 7.2$, all partial $\eta^2 > 0.4$, all $p < 0.0006$, Fig. S3 A-B). As expected, in perturbed trials, movement times were longer than in unperturbed conditions (all $p < 0.03$). No other effects were significant (all $F < 3$, all partial $\eta^2 < 0.17$, all $p > 0.07$). Furthermore, no significant effects on reaction times were observed (all $F < 2.2$, all partial $\eta^2 < 0.13$, all $p > 0.1$).

These analyses confirm the validity of our task design by showing that our pulling machine was effective in producing corrections involving earlier and stronger forearm responses. We also found mild and delayed EMG activations in visual perturbations, a result that aligns with the differing processing speeds of proprioceptive versus visual information [8,9]. As expected, no TMS effects were observed, since in neither experiment we targeted primary motor regions. Additionally, movement times were slower in perturbed conditions due to the delays induced by online corrections.

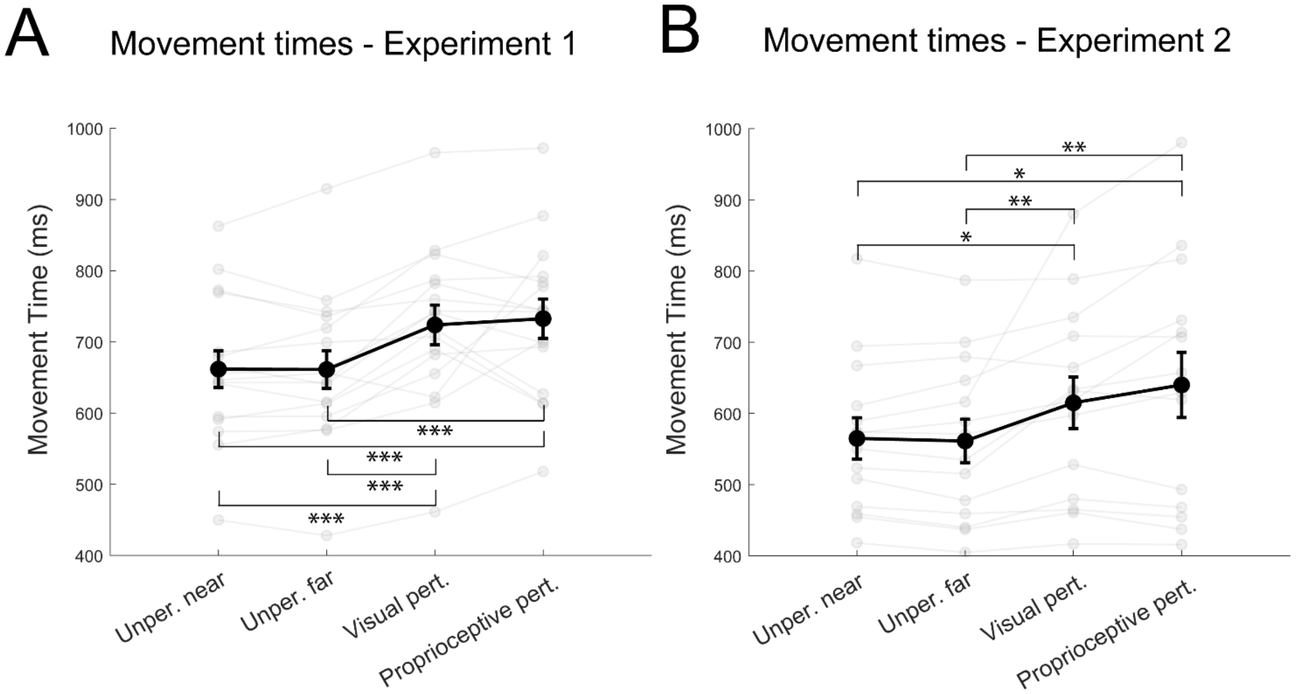

Figure S3. Movement times for both experiments: (A) Results from Experiment 1 (B) Results from Experiment 2. Asterisks mark a statistically significant difference (* = $p < 0.05$, ** = $p < 0.01$, *** = $p < 0.001$). Background lines indicate single subject's data. Unper.near = unperturbed trials towards the near target; Unper.far = unperturbed trials towards the far target; Visual pert = visually perturbed trials; Proprioceptive pert = proprioceptively driven perturbed trials. As no TMS effects were significant, we have collapsed the data from the different stimulation conditions.

Stimulation of parietal and occipital areas affected neither unperturbed reaching movements nor reaching accuracy.

Stimulation of either hV6A, IPS, hPEc, or V1/V2 did not affect the trajectories (ED) of the unperturbed conditions (all $p > 0.15$, see Table S1 and Fig. S4).

No significant main effects or interactions were observed for reaching accuracy either in perturbed or in unperturbed trials (all $F < 2.1$, all partial partial $\eta^2 < 0.12$, all $p > 0.1$), and this suggests that the outcome of the movement was unaffected by the stimulation, as expected given the absence of any stimulation effects in the ED of the index finger at the end of the movement (last bins in Figs. 3-4).

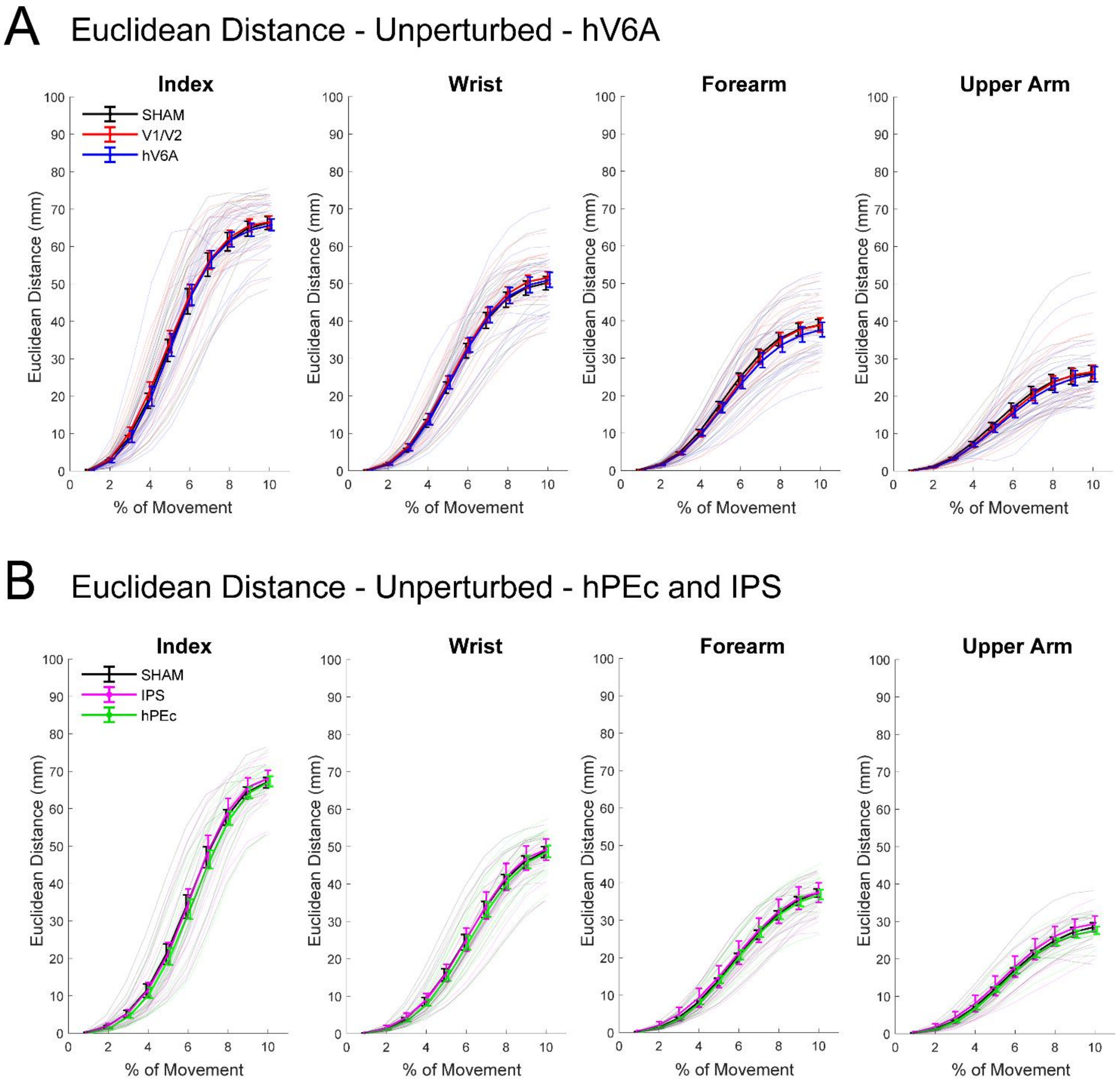

Figure S4. Euclidean distances results for the unperturbed conditions: (A) Results of Experiment 1; (B) Results of Experiment 2. ED at each time bin between trajectories from the unperturbed near condition and the unperturbed far condition in each stimulation condition. No significant differences between stimulation conditions were found. All other conventions as in Figs. 3-4.

	STATIC											
	EXPERIMENT 1						EXPERIMENT 2					
**INDEX**	SS	DOF	MS	F	p	partial η^2	SS	DOF	MS	F	p	partial η^2
TMS	128.8	2	64.4	0.86	0.43	0.05	85.1	2	42.5	0.48	0.62	0.04
Error	2236.7	30	74.6				2291.6	26	88.1			
Bin	300998.9	9	33444.3	428.14	**<0.001**	0.97	270465	9	30051.7	478.56	**<0.001**	0.97
Error	10545.7	135	78.1				7347.1	117	62.8			
TMS*bin	84.9	18	4.7	0.56	0.93	0.04	36	18	2	0.26	0.99	0.02
Error	2272.9	270	8.4				1768	234	7.6			

WRIST	SS	DOF	MS	F	p	partial η^2	SS	DOF	MS	F	p	partial η^2
TMS	128.4	2	64.2	1.06	0.36	0.07	49.5	2	24.8	0.22	0.80	0.02
Error	1820.5	30	60.7				2867.4	26	110.3			
Bin	177279.2	9	19697.7	470.99	**<0.001**	0.97	136598.2	9	15177.6	396.34	**<0.001**	1
Error	5645.9	135	41.8				4480.4	117	38.3			
TMS*bin	31.7	18	1.8	0.37	0.99	0.02	10	18	0.6	0.13	0.99	0.01
Error	1278.2	270	4.7				1037.4	234	4.4			
FOREARM	SS	DOF	MS	F	p	partial η^2	SS	DOF	MS	F	p	partial η^2
TMS	135.94	2	67.97	1.99	0.15	0.12	70.4	2	35.2	0.19	0.83	0.01
Error	1194.2	30	39.8				4766.5	26	183.3			
Bin	37907.22	9	4211.91	27.41	**<0.001**	0.65	76854.6	9	8539.4	436.92	0	1
Error	4194.6	135	31.1				2286.7	117	19.5			
TMS*bin	138.57	18	7.7	0.80	0.70	0.05	21.3	18	1.2	0.27	0.99	0.02
Error	791.4	270	2.9				1029.1	234	4.4			
UPPER ARM	SS	DOF	MS	F	p	partial η^2	SS	DOF	MS	F	p	partial η^2
TMS	11.79	2	5.89	0.25	0.78	0.02	107.73	2	53.87	0.32	0.73	0.02
Error	696.17	30	23.21				4437.54	26	170.67			
Bin	44771.27	9	4974.59	133.90	**<0.001**	0.90	45278.38	9	5030.93	402.39	**<0.001**	1
Error	5015.29	135	37.15				1462.81	117	12.5			
TMS*bin	18.71	18	1.04	0.63	0.87	0.04	24.31	18	1.35	0.31	0.99	0.02
Error	442.28	270	1.64				1027.72	234	4.39			

Table S1. ANOVA results for the ED during the unperturbed conditions. Same conventions as in Table 1 and 2.

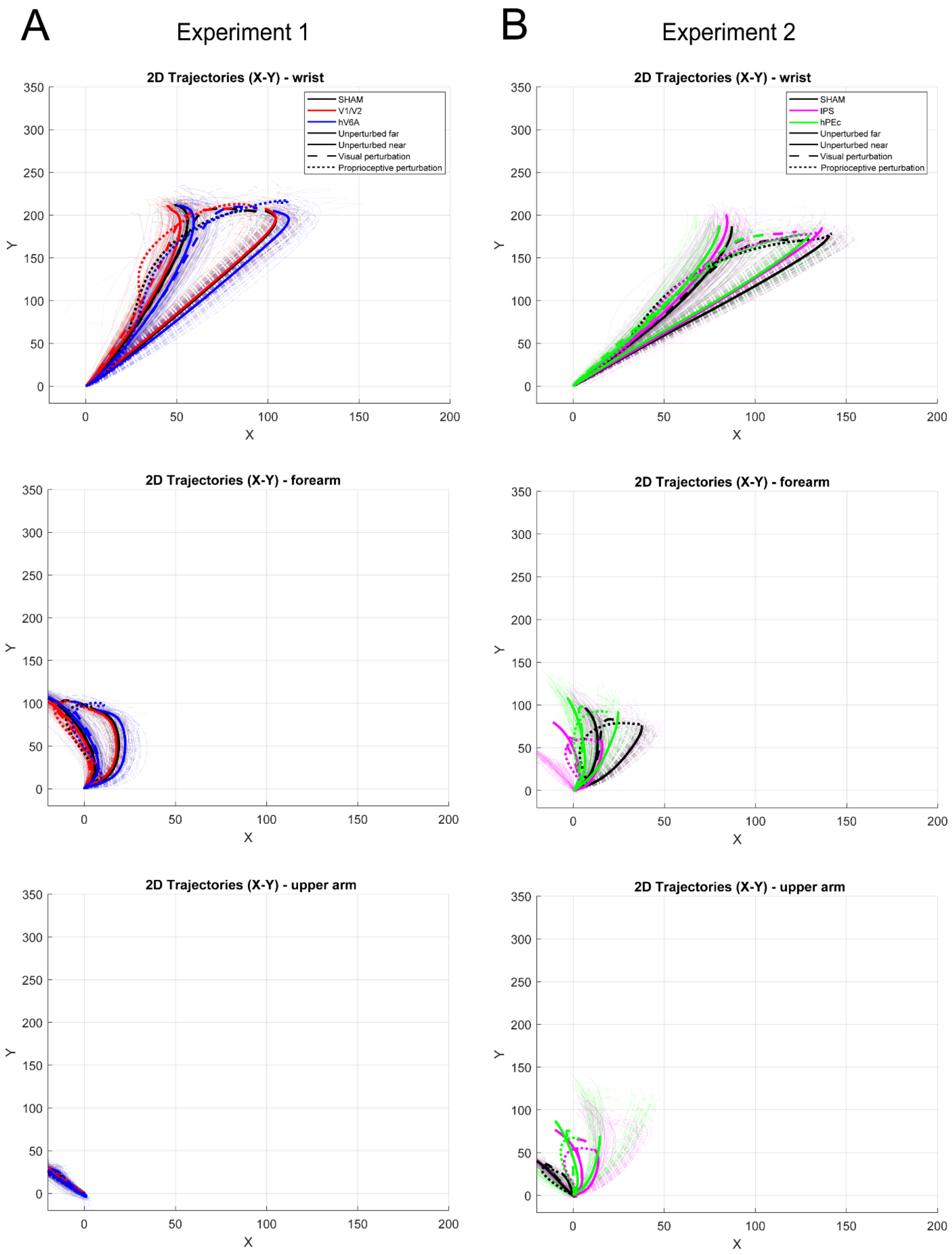

Figure S5. Kinematic data of wrist, forearm and upper arm markers of a representative participant: Two-dimensional plots showing single-trial wrist, forearm and upper arm trajectories (thin lines) along with their averages (thick lines) of two representative participants (one for each experiment) under each stimulation condition for (A) Experiment 1 and (B) Experiment 2.

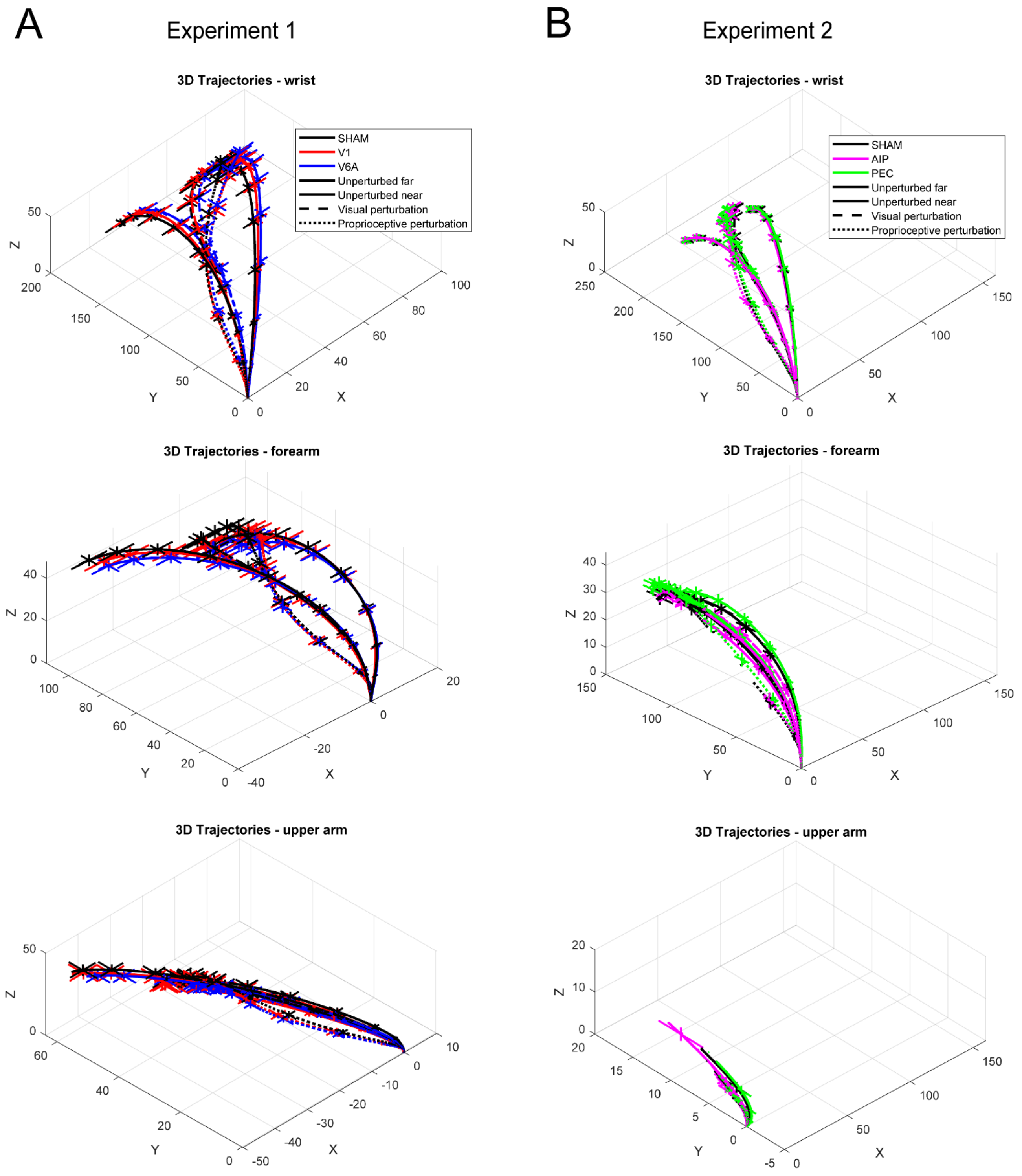

Figure S6. Population trajectories of the wrist, forearm and upper arm. Other conventions as in Figure 5 and S5.

References

1. Rossini, P. M. *et al.* Non-invasive electrical and magnetic stimulation of the brain, spinal cord, roots and peripheral nerves: Basic principles and procedures for routine clinical and research application. An updated report from an I.F.C.N. Committee. *Clin Neurophysiol* **126**, 1071–1107 (2015).

2. Brinkworth, R. S. A. & Türker, K. S. A method for quantifying reflex responses from intra-muscular and surface electromyogram. *J Neurosci Methods* **122**, 179–193 (2003).

3. Ellaway, P. H. Cumulative sum technique and its application to the analysis of peristimulus time histograms. *Electroencephalogr Clin Neurophysiol* **45**, 302–304 (1978).

4. Hadjidimitrakis, K., Moschovakis, A. K., Dalezios, Y. & Grantyn, A. Eye position modulates the electromyographic responses of neck muscles to electrical stimulation of the superior colliculus in the alert cat. *Exp Brain Res* **179**, 1–16 (2007).

5. Breveglieri, R. *et al.* Modulation of reaching by spatial attention. *Front Integr Neurosci* **18**, (2024).

6. Breveglieri, R. *et al.* Complementary contribution of the medial and lateral human parietal cortex to grasping: a repetitive TMS study. *Cereb Cortex* (2022) doi:10.1093/cercor/bhac404.

7. Mahon, A., Bendžiūtė, S., Hesse, C. & Hunt, A. R. Shared attention for action selection and action monitoring in goal-directed reaching. *Psychol Res* **84**, 313–326 (2020).

8. Omrani, M., Pruszynski, J. A., Murnaghan, C. D. & Scott, S. H. Perturbation-evoked responses in primary motor cortex are modulated by behavioral context. *J Neurophysiol* **112**, 2985–3000 (2014).

9. Cecala, A. L., Kozak, R. A., Pruszynski, J. A. & Corneil, B. D. Done in 65 ms: Express Visuomotor Responses in Upper Limb Muscles in Rhesus Macaques. *eNeuro* **10**, ENEURO.0078-23.2023 (2023).
