# Supplementary material for: Functional specialization of the human posterior parietal cortex in visually and proprioceptively driven reaching corrections
Source: Commun Biol. 2025 Nov 24;8:1658. doi: 10.1038/s42003-025-09040-5 (PMC12644796; doi:10.1038/s42003-025-09040-5)
Supplement: Supplementary file 2 — Description of Additional Supplementary Files [file 42003_2025_9040_MOESM2_ESM.docx]

Description of Additional Supplementary Files

**File name:** Supplementary Data

**Description:** Supplementary numerical source data for graphs of the main figures.
