# Supplementary material for: Functional specialization of the human posterior parietal cortex in visually and proprioceptively driven reaching corrections
Source: Commun Biol. 2025 Nov 24;8:1658. doi: 10.1038/s42003-025-09040-5 (PMC12644796; doi:10.1038/s42003-025-09040-5)
Supplement: Supplementary file 4 — Reporting summary [file 42003_2025_9040_MOESM4_ESM.pdf]

nature portfolio

Corresponding author(s): Rossella Breveglieri

Last updated by author(s): Sep 30, 2025

Reporting Summary

Nature Portfolio wishes to improve the reproducibility of the work that we publish. This form provides structure for consistency and transparency in reporting. For further information on Nature Portfolio policies, see our Editorial Policies and the Editorial Policy Checklist.

Statistics

For all statistical analyses, confirm that the following items are present in the figure legend, table legend, main text, or Methods section.

n/a	Confirmed	
☐	☒	The exact sample size (*n*) for each experimental group/condition, given as a discrete number and unit of measurement
☐	☒	A statement on whether measurements were taken from distinct samples or whether the same sample was measured repeatedly
☐	☒	The statistical test(s) used AND whether they are one- or two-sided<br>*Only common tests should be described solely by name; describe more complex techniques in the Methods section.*
☐	☒	A description of all covariates tested
☐	☒	A description of any assumptions or corrections, such as tests of normality and adjustment for multiple comparisons
☐	☒	A full description of the statistical parameters including central tendency (e.g. means) or other basic estimates (e.g. regression coefficient) AND variation (e.g. standard deviation) or associated estimates of uncertainty (e.g. confidence intervals)
☐	☒	For null hypothesis testing, the test statistic (e.g. *F*, *t*, *r*) with confidence intervals, effect sizes, degrees of freedom and *P* value noted<br>*Give P values as exact values whenever suitable.*
☒	☐	For Bayesian analysis, information on the choice of priors and Markov chain Monte Carlo settings
☒	☐	For hierarchical and complex designs, identification of the appropriate level for tests and full reporting of outcomes
☐	☒	Estimates of effect sizes (e.g. Cohen's *d*, Pearson's *r*), indicating how they were calculated

Our web collection on statistics for biologists contains articles on many of the points above.

Software and code

Policy information about availability of computer code

Data collection	MATLAB
Data analysis	MATLAB, STATISTICA

For manuscripts utilizing custom algorithms or software that are central to the research but not yet described in published literature, software must be made available to editors and reviewers. We strongly encourage code deposition in a community repository (e.g. GitHub). See the Nature Portfolio guidelines for submitting code & software for further information.

Data

Policy information about availability of data

All manuscripts must include a data availability statement. This statement should provide the following information, where applicable:

- Accession codes, unique identifiers, or web links for publicly available datasets
- A description of any restrictions on data availability
- For clinical datasets or third party data, please ensure that the statement adheres to our policy

The datasets generated and/or analyzed during the current study are not publicly available because the informed consent signed by the volunteers enrolled in the study did not contain the possibility to share the data publicly. Nevertheless, data is available from the corresponding author upon reasonable request.

Research involving human participants, their data, or biological material

Policy information about studies with human participants or human data. See also policy information about sex, gender (identity/presentation), and sexual orientation and race, ethnicity and racism.

Reporting on sex and gender	RESULTS APPLY TO BOTH SEXES AND GENDERS<br>SEX AND GENDER OF THE PARTICIPANTS WERE DETEMINED BASED ON SELF-REPORTING
Reporting on race, ethnicity, or other socially relevant groupings	NOT APPLICABLE IN OUR MANUSCRIPT
Population characteristics	Thirty healthy adult participants were involved in this study. They were divided into two groups: one group of sixteen took part in the first experiment (average age 23.69 ± 3.77, age range: 19-32, 2 males); another group of fourteen took part in the second experiment (average age 24.86 ± 3.46, age range: 20-32, 5 males).
Recruitment	PARTICIPANTS WERE RECRUITED WITH ANNOUNCEMENTS PUT IN THE UNIVERSITY BUILDINGS.
Ethics oversight	COMITATO DI BIOETICA, UNIVERSITA' DI BOLOGNA (ITALY)

Note that full information on the approval of the study protocol must also be provided in the manuscript.

Field-specific reporting

Please select the one below that is the best fit for your research. If you are not sure, read the appropriate sections before making your selection.

☒ Life sciences ☐ Behavioural & social sciences ☐ Ecological, evolutionary & environmental sciences

For a reference copy of the document with all sections, see nature.com/documents/nr-reporting-summary-flat.pdf

Life sciences study design

All studies must disclose on these points even when the disclosure is negative.

Sample size	A POWER ANALYSIS WAS PERFORMED (GPOWER SOFTWARE) FOR EACH OF THE TWO EXPERIMENTS. MOREOVER, THE SAMPLE SIZE USED HERE WAS IN KEEPING WITH OTHER STUDIES IN THE RELEVANT LITERATURE.
Data exclusions	TWO PARTICIPANTS WERE EXCLUDED FROM THE EXPERIMENT 2 BECAUSE OF TECHNICAL ISSUES (the analysis presented in the paper were performed on the remaining 30 participants)
Replication	Robust statistical tests were performed and described in this paper, and this should ensure reproducibility of the results in future studies. sample size (number of participants and trials) were appropriate to ensure reproducibility of the results.
Randomization	THE PARTICIPANTS WERE RANDOMLY ASSIGNED TO EACH EXPERIMENT.
Blinding	INVESTIGATORS HAD TO KNOW WHERE TO PUT THE COIL OVER THE BRAIN DURING THE EXPERIMENTS, SO BLINDING WAS NOT POSSIBLE (AS IN ALL THE TMS STUDIES IN THE LITERATURE).

Reporting for specific materials, systems and methods

We require information from authors about some types of materials, experimental systems and methods used in many studies. Here, indicate whether each material, system or method listed is relevant to your study. If you are not sure if a list item applies to your research, read the appropriate section before selecting a response.

Materials & experimental systems

n/a	Involved in the study	
☒	☐	Antibodies
☒	☐	Eukaryotic cell lines
☒	☐	Palaeontology and archaeology
☒	☐	Animals and other organisms
☒	☐	Clinical data
☒	☐	Dual use research of concern
☒	☐	Plants

Methods

n/a	Involved in the study	
☒	☐	ChIP-seq
☒	☐	Flow cytometry
☒	☐	MRI-based neuroimaging

Plants

Seed stocks	n/a
Novel plant genotypes	n/a
Authentication	n/a
